# RF-induced heating of interventional devices at 23.66 MHz

**DOI:** 10.1007/s10334-023-01099-7

**Published:** 2023-05-17

**Authors:** Ali Caglar Özen, Maximilian Frederik Russe, Thomas Lottner, Simon Reiss, Sebastian Littin, Maxim Zaitsev, Michael Bock

**Affiliations:** 1 Division of Medical Physics, Department of Diagnostic and Interventional Radiology, University Medical Center Freiburg, Faculty of Medicine, University of Freiburg, Freiburg, Germany; 2 Department of Diagnostic and Interventional Radiology, University Medical Center Freiburg, Faculty of Medicine, University of Freiburg, Freiburg, Germany

**Keywords:** Magnetic resonance imaging, RF-induced heating, MR safety, Interventional MRI, MR-guided intervention, Cardiovascular catheterization, Hepatic artery catheterization, Low field MRI

## Abstract

**Objective:**

Low-field MRI systems are expected to cause less RF heating in conventional interventional devices due to lower Larmor frequency. We systematically evaluate RF-induced heating of commonly used intravascular devices at the Larmor frequency of a 0.55 T system (23.66 MHz) with a focus on the effect of patient size, target organ, and device position on maximum temperature rise.

**Materials and methods:**

To assess RF-induced heating, high-resolution measurements of the electric field, temperature, and transfer function were combined. Realistic device trajectories were derived from vascular models to evaluate the variation of the temperature increase as a function of the device trajectory. At a low-field RF test bench, the effects of patient size and positioning, target organ (liver and heart) and body coil type were measured for six commonly used interventional devices (two guidewires, two catheters, an applicator and a biopsy needle).

**Results:**

Electric field mapping shows that the hotspots are not necessarily localized at the device tip. Of all procedures, the liver catheterizations showed the lowest heating, and a modification of the transmit body coil could further reduce the temperature increase. For common commercial needles no significant heating was measured at the needle tip. Comparable local SAR values were found in the temperature measurements and the TF-based calculations.

**Conclusion:**

At low fields, interventions with shorter insertion lengths such as hepatic catheterizations result in less RF-induced heating than coronary interventions. The maximum temperature increase depends on body coil design.

**Supplementary Information:**

The online version contains supplementary material available at 10.1007/s10334-023-01099-7.

## Introduction

Magnetic resonance imaging (MRI)-guided intravascular operations were introduced in early 2000s [1–5]. With the advances in imaging methods [6–8] and active marker technologies [9–13], MRI-guided cardiac [14–21] and hepatic artery catheterization or percutaneous interventions [22–28] have been performed. MRI-guided procedures offer numerous advantages over conventional X-ray-guided interventions including radiation-free 3D soft tissue visualization and functional hemodynamic data; however, the clinical translation has been limited by the lack of the availability of MR-compatible devices [29]. Instead of adapting the devices to the MRI environment, another approach is making the MRI “device-compatible” by using low SAR protocols [30], using parallel transmission technologies [31, 32], real-time control of radiofrequency transmission [33], or using low-field systems which operate at lower frequency [34].

A particular safety concern is radiofrequency-(RF)-induced heating of implants and devices in the magnetic resonance imaging (MRI) environment as a result of the coupling of the applied electromagnetic (EM) transmit fields with elongated metallic structures [35, 36]. During the RF transmission, the EM energy is coupled to the conducting metallic structures (e.g., implant leads or guidewires) where it is often deposited at a hotspot. Typically, hotspots are located at the end of the structure, for example close to the tip of the electrodes in implanted neurostimulator systems, at the end of a guidewire, or at the tip of a needle. The resulting heating depends on the incident E-field generated by the transmit coil [37], the EM properties of both the implant/device and the surrounding tissue, the input impedance [33, 38] and the device insertion length [39].

Compared to current clinical MRI systems at 1.5 T or 3 T, low-field MRI systems (e.g., at 0.55 T) are expected to cause less RF-induced heating as they operate at lower Larmor frequencies which are associated with longer wavelengths [34]. Even though the advantages of low-field MRI have been shown for some devices, a systematic assessment of the RF-induced heating of interventional devices has so far not been performed. In general, the guidelines to assess RF-induced heating of devices and implants are based on two approaches: measurements of the temperature increase around implants for an empirically chosen worst-case position [40], or the analysis of RF exposure along the lead pathways in combination with a measurement of the position-independent lead RF response [41].

The first approach, as proposed by the American Society for Testing and Materials (ASTM), covers measurement of RF-induced heating during MRI on or near a passive medical implant within a tissue-simulating phantom [40]. The second, so-called domain decomposition approach as described in Tier-3 Clause #8 of ISO 10974 Ed2 [41] analyses the RF exposure by the MRI RF transmit coil using a set of clinically relevant incident tangential RF electric fields (*E*_tan_(*z*)). These tangential fields are then propagated along the lead or wire paths using a position-independent RF response of the device, the so-called transfer function (TF). Although the ISO 10974 standard is intended for active implantable medical devices, in principle, it can also be applied to interventional guiding catheters (GC), guidewires (GW) and biopsy needles (BN). During an intervention the devices are only partially immersed in the body, and their insertion length varies with time. Moreover, the temperature hotspot is not necessarily located at an exposed tip; thus, the actual hotspots need to be located prior to safety evaluations based on TF or local temperature measurements.

In this study, we combine high-resolution electric field mapping for hotspot detection with transfer function (TF) measurements to assess RF-induced heating of four commonly used intravascular devices, and we perform temperature measurements on an applicator and a biopsy needle. We use realistic vascular models to imitate device trajectories in the human body, and compare RF-induced heating of the intravascular devices for different patient sizes, different target organs, two RF body coils, and for various positions.

## Methods

**Fig. 1:**
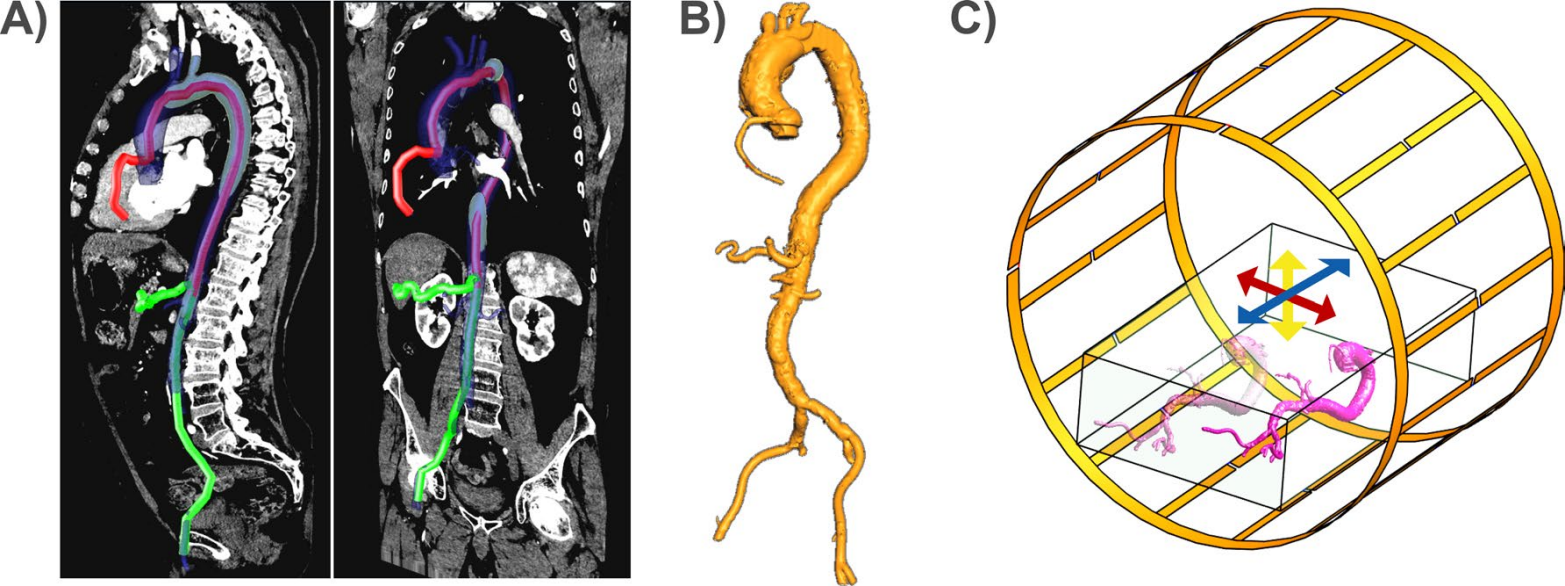
**A** Vascular models were extracted from CT angiograms of two patients (157 cm, Female and 185 cm, Male). Coronal and sagittal slices form the angiograms show the differences in insertion lengths for hepatic artery (HA, green-colored track), and coronary artery (CA, red-colored track) engagement, starting with a femoral access. **B** A 3D-printed vascular model to guide the devices and to form the realistic trajectories during RF-induced heating tests. **C** 3D models of Coil 1, ASTM phantom, and the vascular model. 3D-printed models were fixed inside the ASTM phantom and the position was varied in each direction

We selected hepatic artery (HA) and coronary artery (CA) interventions as test cases as they are frequently applied in clinical practice and present distinct device trajectories and insertion lengths. To derive realistic trajectories, CT angiograms of two patients (157 cm, F; 185 cm, M) were segmented to create vascular models that were constructed by 3D-printing. The models ranged from the femoral artery access points via the HA to the left and right CA (Fig. 1).

For safety assessment in this work, a modified Tier-3 approach [42] was applied. High-resolution E-field maps were measured for hotspot detection during RF excitation at *f*_Larmor_(0.55 T) = 23.66 MHz followed by TF measurements at the hotspot [39]. The following clinical-routine devices were tested: Terumo Radiofocus Guidewire (GW) M with *Ø* = 0.89 mm, length = 180 cm (Terumo Europe E.V., Leuven, Belgium) and Medtronic SiteSeer 5F guiding catheter (GC) with *Ø* = 1.67 mm, length = 100 cm (Medtronic Intl. Trading SARL, Tolochenaz, Switzerland) for CA engagement; and Radiofocus Guidewire M with *Ø* = 0.89 mm, length = 260 cm, straight tip (Terumo) and a microcatheter (μC) with *Ø* = 0.94 mm and length = 110 cm (Terumo) for HA engagement.

### Hotspot detection

**Fig. 2:**
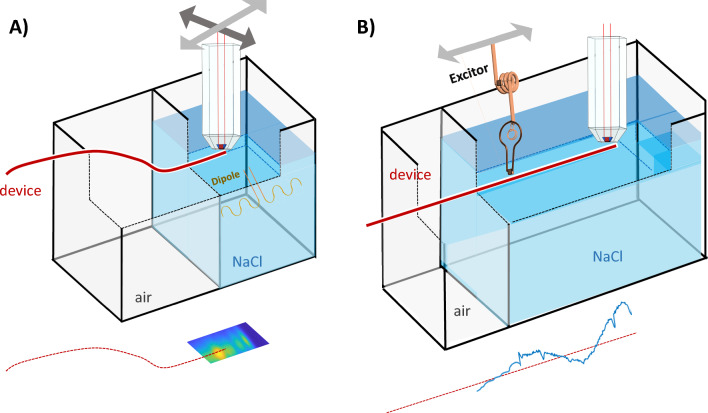
Schematic of the hotspot detection and TF evaluation setups for the intravascular devices. **A** Hotspot detection with high-resolution E-field mapping during continuous RF excitation with a dipole antenna. **B** TF measurement. A detailed schematic of the electro-optical sensor (EOS)

The first step in the safety evaluation was the detection of the hotspot near the device. Therefore, devices were attached to a straight sample holder and immersed in a 2-compartment phantom (container size 30 × 40 × 140 cm^3^, Fig. 2) which allows to partially insert an elongated device in a bath filled with physiologic saline solution (${\epsilon }_{r}=81, \sigma =0.55$ S/m). For RF excitation, an 80 cm long meandered dipole antenna was positioned inside the bath (*S*_11_(23.66 MHz) =  − 10.5 dB) parallel to the device at a distance of 8 cm with the end of the dipole aligned with the device tip. The dipole was isolated by a water-tight silicon putty previously used for covering intraoral coils [43].

To map the E-field, an electro-optic sensor (EOS) was used based on the Pockel’s effect, where the refractive index of an optical crystal changes linearly with the external electric field [44]. In this EOS, the polarization angle of a 1330 nm probe laser (Orion, Rio Redfern Integrated Optics, Santa Clara, CA) beam is shifted depending on the strength of the external E-field penetrating the crystal. The EOS consists of a LiNbO_3_ electro-optical crystal (1 × 1 × 1 mm^3^) which is sensing the normal component of the E-field with respect to the laser beam. Before entering the EOS crystal, a linear polarization of the laser beam is defined using a half-wave plate, and two right-angle prisms are applied to guide the beam through the crystal where the E-field-dependent rotation of the polarization takes place. After the crystal, the beam is passing through a quarter-wave plate and a fiber-based polarization beam splitter from which the two polarization components of the beam are guided to a balanced photo detector (PDB480C-AC, Thorlabs Inc., Newton, New Jersey). The detector provides the AC-coupled difference of the two components as output, which is linearly dependent on the E-field strength [39, 45, 46]. A lock-in amplifier (UHFLI, Zürich Instruments, Zürich, Switzerland) with an integrated waveform generator is used for generating the excitation signal as well as for the demodulation and digitization of the EOS signal.

Prior to the measurements in the phantom, the EOS was calibrated with a TEM cell (TBTC2 Open TEM Cell, TekBox Digital Solutions, Vietnam). In the subsequent phantom measurements, a motor-driven 2D stage was used for an automatic positioning of the EOS. E-field maps were measured 2 mm above the devices for the two patient sizes (small / large with insertion lengths of 65 cm/80 cm) and the two interventional scenarios (coronary artery/hepatic artery). Once the hotspot was found for each scenario, the EOS was positioned at this location for the TF measurement.

### TF measurements

In the TF measurements, the same 2D motor stage was used as in the E-field mapping to move an excitor along the device, while the EOS was fixed at the hotspot (Fig. 2B). For the measurement, two excitors were built for the air and the saline solution compartment; the excitors were constructed as inductively coupled loop coils tuned to *f*_Larmor_ depending on the loading conditions in air and saline solution (NaCl) using a single multi-layer ceramic capacitor. The excitors created a localized tangential E-field along the device [39] and were moved along the device in 2 mm steps, while E-field was measured with the EOS to create the TF.

In addition, background measurements without the device were acquired and subtracted from the measurements with the device. To validate the TF, two different dipole antennae were used to generate a distributed E-field [42]. The E-field was first mapped without the device, and the scattered E-field at the hotspot was calculated by integrating the tangential component of the E-field along the device ***E***_tan_(*z*) using the TF [37]. Then, for comparison, the scattered E-field was measured using the EOS at the hotspot.

### Temperature measurements

**Fig. 3:**
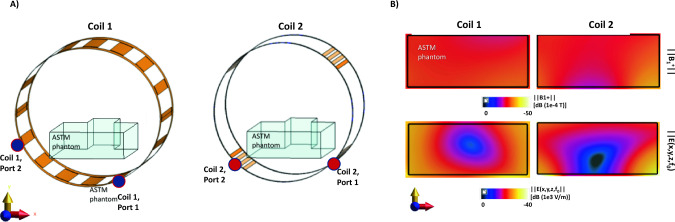
**A** A schematic displaying the feed-port locations for both coils. **B** |***B***_1_ +| and |***E***(*x*,*y*,*z*)| field maps obtained from FDTD simulations for 1 kW input power. The major differences between the coils are the location of the feed ports and the length of the RF shield around them. The E-fields were extracted for a mean |***B***_1_ +| of 10uT at the center of the ASTM model. The peak |***B***_1_ +| magnitude was also verified during the measurements using a magnetic field probe located at the isocenter of the coils

An RF test bench was constructed for temperature measurements with a standard quadrature body coil (Coil1: *Ø* = 790 mm, Length = 500 mm) and a modified 16-leg-birdcage coil (Coil2: *Ø* = 800 mm, Length = 500 mm). Coil 2 had an asymmetric distribution of the rungs, where groups of four rungs were distributed at 45°, 135°, 225°, and 315° angles in azimuthal direction [47]. The feed ports were located at –22.5°/67.5° for Coil 1 and at –45°/45° for Coil 2, respectively (Fig. 3). Endrings of Coil 2 had a slightly larger diameter of 820 mm than the cylindrical volume formed by the rungs (*Ø* = 800 mm). In Fig. 3B, the simulated |***B***_1_^+^| and |***E***| field maps of both coils are shown within the tissue-simulating ASTM phantom for an input power of 1 kW. Both body coils were provided by Siemens Healthineers.

During the temperature measurements at the test bench, the coils were actively tuned (*U*_DC_ = 5 V at *I*_DC_ = 1.05A) using a four-channel DC supply (HMP4040, HAMEG, Mainhausen, Germany). A function generator (HM 8130, HAMEG) was used to trigger an RF signal generator (EXG N5171B, Keysight, Colorado Springs, CO) at a duty cycle of 20%, resembling a rectangular RF pulse of 1 ms duration at a TR of 5 ms. The output of the signal generator was connected to a broadband RF power amplifier (RFPA, Siemens, Erlangen, Germany), which can generate up to 2 kW continuous wave power. The coils were driven in quadrature mode using a hybrid coupler (HYBRID M05 D04, Quality Electrodynamics, Mayfield Village, OH) in which the isolation port was terminated by a 50 Ω dummy load with 200 W power rating. The output of the RFPA was continuously monitored using a 30 dB-directional coupler (EME 7020–30 A, UKW-Berichte Telecommunications, Eggolsheim, Germany) connected to an oscilloscope (MSO 4104, Tektronix, Beaverton, OR). The input power was adjusted using a calibrated *B*_1_-probe (2.5 cm diameter loop coil made of semi-rigid coaxial cable) which was connected to a spectrum analyzer (N9320B, Keysight). The RF power of the signal generator was set to the same power level as that of an 180° RF pulse measured at a prototype magnet using the same body coils.

Temperature measurements were performed at different positions of the phantom and the vascular model by shifting them in *x*- and *y*-direction by ± 10 cm, and in z-direction by ± 5 cm (Fig. 1C). Different cardiac catheterization methods (femoral artery right/left access) and insertion lengths (80, 85, 90, 95 cm) as well as patient (head/feet-first) and operator (left/right) positions were assessed for the GW. For HA catheterization, insertion lengths of 60, 65, 70 cm were tested. RF heating was measured using a pulse train (pulse duration: 1 ms, TR = 5 ms, power: 1.8 W/kg, 2-min heating).

In the abdomen, also percutaneous interventions are commonly performed. Therefore, additional temperature measurements were acquired for an applicator with a coaxial BN pair: 15/20 cm, and an introducer needle: 7.5/10 cm (Amica Probe, HS Hospital Service, Rome, Italy) for various insertion lengths and positions (Supporting Information Fig. S1).

### Electro-magnetic simulations

Body coils were simulated using a finite‐difference time‐domain (FDTD) solver (Sim4Life v7.0, ZMT, Zürich), and tangential E-fields along the trajectories were extracted to calculate temperature rise based on TF measurements. S-parameters for both coils were matched approximately to the measured values by fine-tuning of feed port and isolation capacitors. For Coil 1, measured and simulated S-parameters, (*S*_11_, *S*_22_, *S*_21_), were (− 8.9, − 9.3, − 17.6) dB, and (− 9.4, − 10.0, − 15.9) dB, respectively; and for Coil2, (− 10.5, − 9.4, − 15.6) dB, and (− 10.3, − 9.7, − 14.2) dB. Coil conductors were modeled as perfect electric conductors (PEC). A gel phantom model was introduced as load with *ε*_r_ = 80, *σ* = 0.6 S/m at *f*_Larmor_. The coils were driven in quadrature mode with 90° phase difference between the ports. Each body coil simulation consisted of 4.7 million voxels, where the minimum voxel size was 2 × 4 × 4 mm^3^. Computations were performed using a graphical processing unit (NVidia Tesla P100, NVIDIA Corporation, Santa Clara, US). For extraction of*** E***_tan_(*z*), the RF input power was scaled to achieve a mean |***B***_1_^+^| of 11.75 µT within the center of the phantom.

Details of the simulation settings are provided in supplementary material. 3D models and capacitor values of a generic birdcage coil, phantom, vascular models and the Python scripts used in analysis of the simulation results can be downloaded from https://github.com/ozenEEE/UKF_SafeLowB0.

## Results

**Fig. 4:**
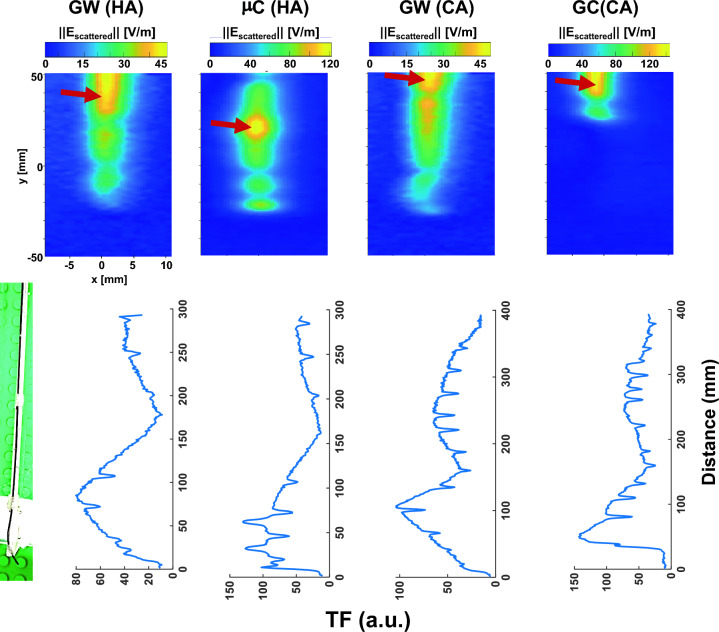
(Upper row) E-field maps of the devices displayed on a 20 × 100 mm^2^ grid with 1 mm resolution. Hotspots location (indicated by red arrow) can even be far away from the tip as the guidewires and some of the catheters have irregular wiring patterns to meet mechanical requirements during interventional operations. (Bottom row) TF measurements performed using piecewise excitation method with an electro-optical sensor

The electric field maps acquired with the EOS show that hotspots do not necessarily occur at the device tip but can be at distances of up to 7.25 cm away from the tip as is shown for the GW (CA) in Fig. 4. The location of the hotspot did not change for different insertion lengths. The peak magnitude of the scattered E-field was higher for the catheters than for the guidewires, with 73, 129, 97, and 145 V/m for GW and μC for HA, and GW and GC for CA interventions, respectively. Measured TFs are shown for all four devices for the first 30 and 40 cm section of the devices (Fig. 4, bottom row). Various oscillating patterns are observed in the TFs which can be attributed to the braiding and wiring patterns in the devices.

**Fig. 5:**
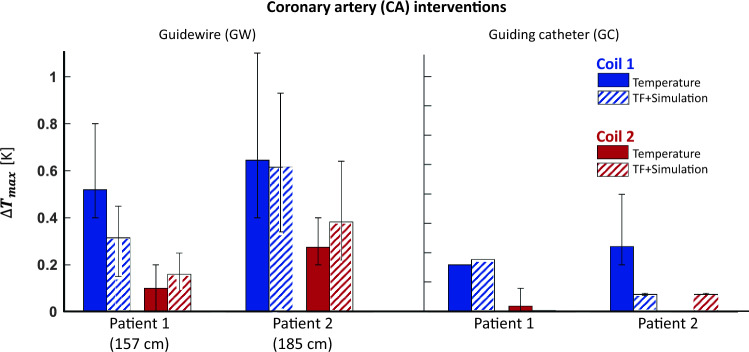
Temperature measurements for coronary artery (CA) case. RF-induced heating tests were performed at different positions of the vascular models inside the phantom. The “error” bars correspond to minimum and maximum temperature rise for all different positions. The selected GW resulted in higher temperature rise than GC for the CA intervention case. Maximum temperature rise for the larger patient is also consistently higher than smaller patient. Coil 2 caused less heating than Coil 1 for all measurements. TF-based temperature rise estimations deviates less than 0.4 K than the measured values

Temperature measurements for the CA interventions are summarized in Fig. 5. A maximum temperature increase of 1.1 K was measured for Coil 1, Patient 2, using the GW. The minimum temperature increase for the same case was 0.4 K, which demonstrates the strong position dependency of the temperature rise. ∆*T*_max_ was consistently higher for Patient 2 by 0.3 K. The difference between ∆*T*_max_ for right and left CA was less than 0.2 K. For the CA case, Coil 2 causes a temperature increase that is up to 0.7 K less than Coil 1. For Coil 1 and the GW intervention on Patient 2, the head-first configuration resulted in 0.4 K less temperature rise compared to feet-first configuration. For Coil 2, however, less than 0.1 K difference was found. Maximum temperature rises for GC was 0.2 K and 0.5 K for Patients 1 and 2, respectively, which is 0.6 K and 0.4 K lower than GW for Coils 1 and 2.

**Fig. 6:**
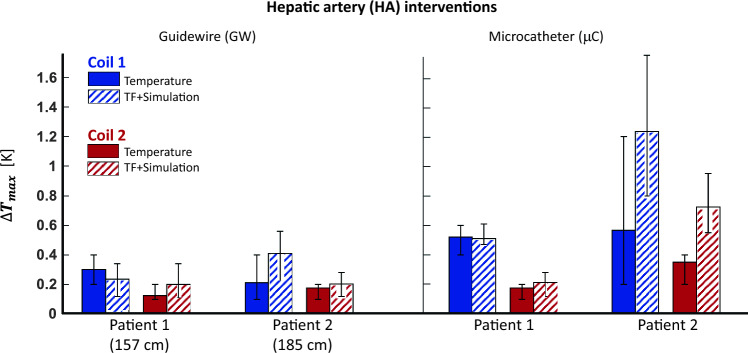
Temperature measurements for hepatic artery (HA) case. RF-induced heating tests were performed at different positions of the vascular models inside the phantom. The “error” bars correspond to minimum and maximum temperature rise for all different positions. The microcatheter (μC) resulted in higher temperature rise than GW. Maximum temperature rise for the larger patient is higher than the smaller patient for μC, yet no significant difference was observed for the GW. Coil 2 caused less heating than Coil 1 except for Patient 1 GW simulations. TF slightly overestimated the temperature rise, except the large patient and mC case, where the temperature rise was overestimated by up to 0.55 K

HA catheterization with the GW resulted in a lower temperature increase (∆*T*_max_ = 0.4 K) than the RCA and LCA trajectories for all patient sizes and positions (Fig. 6). HA trajectories did not vary significantly between two patient models, with Coil 2 resulting in a lower maximum temperature rise by 0.2 K than Coil 1. For the μC, however, the temperature at the hotspot increased by up to 1.2 K and Coil 1 caused up to 0.8 K higher temperature rise than Coil 2. Maximum position-dependent variation was 0.3 K except for μC (1 K of position-dependent variation) compared to 0.7 K for the CA interventions. ∆*T*_max_ for Patient 2 was consistently higher than or equal to Patient 1.

TF-based temperature rise and RF-induced heating measurements were comparable for Coil 2. ∆*T*_max_ was overestimated for all cases with a maximum difference of 0.28 K and 0.55 K for CA and HA interventions, respectively (Figs. 5 and 6). For CA interventions for Coil 1, TF-based maximum temperature rise was underestimated, except for GC and Patient 1 combination. The position-dependent variations were, however, comparable between the TF-based and measured temperature rise. The results of all temperature measurements including TF-based temperature rise estimations are given in Supporting Information Table S1. For the applicator and the needle, a maximum temperature increase of 0.4 K for Coil 1, and of 0.2 K for Coil 2 was measured for all the tested cases.

Although the RF-induced heating could be measured only for 2 min due to the technical limitations of the RF test bench, the temperature curves were already saturated at the end of this period. Local SAR estimated from the temperature curves was 276 W/kg in the most extreme case and was in good agreement with the simulations. Difference in the coupling between feed ports in simulated and measured Coil 2 resulted in deviations from the measured values especially for HA interventions for Patient 1, and CA interventions for Patient 2.

## Discussion

In this study, we introduced an evaluation method for RF-induced heating that involves hotspot detection using high-resolution E-field mapping and TF measurements. We applied this method to evaluate MR safety of four commonly used intravascular devices at *f*_Larmor_. In addition, we measured two different body coils on an RF test bench and performed temperature measurements using realistic device trajectories extracted from angiogram data of two patients with different body sizes.

Hotspot detection is a key factor in safety evaluations as the scattered E-fields can vary by an order of magnitude within the vicinity of the hotspot. Thus, slight displacements of the temperature probe or the E-field sensor in TF measurements might result in large errors. For guiding catheters, the hotspots were close to the point where the braiding ends. Depending on the braiding structure and pattern, it might shift slightly, yet the whole braiding is expected to behave similar to a hollow wire. More details on the effect of the length of the braiding on the temperature rise is studied in [48], and the effect of various braiding patterns on the E-field distribution of stents is investigated in [46]. Guidewires, on the other hand, have a more complex structure, with a core, taper, and a spring coil, and the hotspot has only been experimentally determined based on a manual search with an FOTP [13]. We believe, a direct E-field mapping is more accurate and convenient way of hotspot detection, and is reproducible. The highest temperature rises were measured consistently at the temperature probes located at the hotspot. The accuracy of hotspot detection is limited by the size of the crystal in the EOS. A smaller crystal will be less sensitive to the changes in E-field, yet, provide a higher spatial resolution. MR thermometry (MRT) can be used for hotspot detection, and was shown to be consistent with E-field maps as demonstrated for an intracranial electrode array [49]. However, sensitivity of MRT is lower than the FOTPs or the EOS. MRT can only detect temperature changes with about ± 0.5 K precision. Given the small temperature rise observed at low fields, MRT might not be useful for RF-induced heating evaluations.

HA catheterizations required shorter insertion lengths than CA, leading to a reduced RF-induced heating as shown in both temperature and TF measurements. The resonance length at 23.66 MHz was measured to be 120 cm (Supporting Information Fig. S2), which is above typical device insertion lengths for all studied cases. Thus, our results indicate that an extended safety margin might be applicable at 0.55 T compared to 1.5 T (resonance length: 35 cm). We have shown previously that partially immersed devices do not exhibit the same resonant behavior as in the fully immersed case; however, the partially immersed resonant length was consistently shorter than the fully immersed resonant length [38].

The main advantage of the proposed method is that a single EOS setup can be used for hotspot detection, TF evaluation of the partially immersed device, and validation. The setup could be improved by addition of multiple dipole sources to create circularly polarized incident fields, or by integrating the whole E-field detector bench inside a body Tx coil. Recently, a test setup was suggested which optimizes trajectories with minimal correlation of the incident E-field [50]. As proposed in this study, ***E***_tan_(*z*) is generated by different dipole antennae which are intrinsically uncorrelated; thus, they are ideal for validation measurements. In general, dipole antennae are advantageous for calibration and validation of TFs as they can provide compact E-field sources with variable polarizations which are easy to position without the need to move the device under investigation. Thus, the setup in this study could also be applied to study device safety in other low-field system configurations.

This study highlights also the effect of the body coil on the RF-induced heating. Coil 2 resulted in a lower temperature rises for the same ***B***_1_^+^ consistently for all device and vascular model combinations. This can be attributed to the following differences in the coil designs: Coil 1/Coil 2, low/high-pass; 790/820 mm end-ring diameter; 202.5°&292.5°/225°&315° feed-port locations; 16 equally distributed rungs/16 rungs grouped in 4; distance between the shield and the coil conductors 26/20 mm. Although in this study, Coil 2 resulted in a lower RF-induced heating, this does not exclude the possibility of device trajectory/patient positioning that might yield a different result. A 1.5 T study highlights the differences in high-pass and low-pass birdcage coils for the resulting SAR and E-field distribution [51]. A slightly larger coil diameter is expected to reduce E-fields, and the 20 mm larger end-ring diameter in Coil 2 resembles the bumped dipole antenna approach to move the feed port away from the tissue [52]. As shown in Fig. 3, both coils have a low-E-field region around the center of the ASTM phantom. For linearly polarized birdcage coils, there is a zero E-field plane. This zero E-field plane can be manipulated to minimize coupling as shown in [53, 54], as a geometric decoupling approach. Similarly, the low-E-field region is manipulated by the location of feed ports and respective phases of the feed ports. This and the number of rungs, however, might affect the RF-induced heating in negative or positive way, depending on the device trajectory and patient position. A more extreme case of different body coil types is body coils used in open-sided C-arm or double-donut magnet designs, which has been shown to be a significant factor in RF-induced heating of active and passive medical implants [55–57]. The advantages and disadvantages of open and close-bore magnets were discussed in [58]. Since open-sided magnets offer only up to 47 cm opening, patient positioning and access might be further limited, especially for patients with disabilities or large BMI. Shorter and larger diameter magnets offer better patient access as well as larger imaging FOV. The body coil prototypes used in this study are suitable for a horizontal whole-body close-bore magnet and has an inner diameter of 80 cm. Having a relatively shorter length of 500 mm can further enhance intravascular and percutaneous interventions by improving both patient and operator comfort if the total magnet length can also be kept short. A systematic comparison of the state-of-the-art open and low-field close-bore magnet systems is needed.

The proposed method can also be implemented in a clinical MRI system once the mechanical components are replaced with nonmagnetic counterparts. For example, an MR-compatible translational stage as is used in the E-field measurements was described in [59]. For absolute temperature measurements with GaAs-based fiber optic temperature probes, however, the sensors must be calibrated inside the magnet to take B0-dependent band gap changes into account [60]. For hotspot detection, a dipole antenna was used to generate incident E-fields. Even though a dipole antenna generates a linearly polarized field unlike the quadrature-driven birdcage body coils, they were preferred for their flexibility in integration to the EOS setup. Besides, E-fields generated by dipole antennae can be more efficient than a birdcage coil [61]. In principle, the TF approach eliminates the need for the use of a predetermined field pattern. Clause 8.8 of ISO 10974 justifies both the use of clinically relevant exposure characteristics or of incident field exposures based on model response characteristics for validation of a TF [41]. We also did not observe any hotspot changes with respect to the location of the dipole antenna, although, the change in position of the dipole corresponds to a change in incident field pattern. A systematic study that compares dipoles and birdcage coils in TF validation and hotspot detection will be a part of future studies.

## Conclusion

This study shows that at low magnetic fields of 0.55 T, the RF-induced heating of commonly used intravascular devices depends on the patient size and target organ. Reduced heating is observed for smaller patients and hepatic artery interventions where shorter insertion lengths are needed compared to cardiovascular interventions. The type of body coil is a key factor in the resulting temperature increase; heating can be significantly reduced using RF coils with larger diameters, feed ports located at a larger distance and/or different orientations. For devices with much shorter dimensions than the RF wavelength such as biopsy needles or microwave applicators, we did not observe any significant heating—thus, low-field systems can be considered to be MR-safe for certain devices. Systematic evaluation of other devices and implants, using different patient models and inhomogeneous phantoms, can provide further insights on the potential safety of low-field systems for MR-guided interventions.

## Supplementary Information

Below is the link to the electronic supplementary material.

Supplementary file1 (PDF 907 KB)

## Data Availability

The datasets used and/or analysed during the current study are available from the corresponding author.
