# Supplementary material for: RF-induced heating of interventional devices at 23.66 MHz
Source: MAGMA. 2023 May 17;36(3):439–49. doi: 10.1007/s10334-023-01099-7 (PMC10386938; doi:10.1007/s10334-023-01099-7)
Supplement: Supplementary file 1 — Supplementary file1 (PDF 907 KB) [file 10334_2023_1099_MOESM1_ESM.pdf]

RF-induced heating of interventional devices at 23.7 MHz

Özen AC[1], Russe M[2], Lottner T[1], Reiss S[1], Littin S[1], Zaitsev M[1], Bock M[1]

[1]Division of Medical Physics, [2]Department of Diagnostic and Interventional Radiology, University Medical Center Freiburg, Faculty of Medicine, University of Freiburg, Freiburg, Germany

Short running title: Safe Interventions at Low Fields

Supplementary Material

Supporting Information Figures

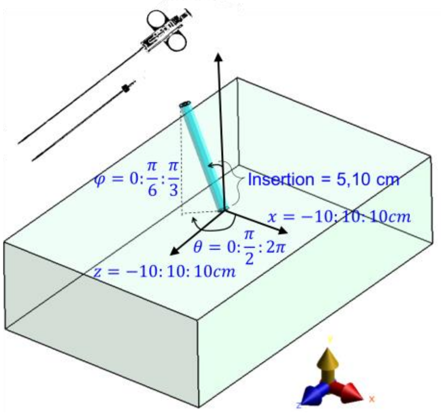

Supporting Information Figure S1: Temperature measurement setup for a coaxial BN pair. The needles were tested for various insertion lengths and positions. The fiber optic temperature probes were placed around the tip of the needles. No significant heating was detected in any of the tested positions.

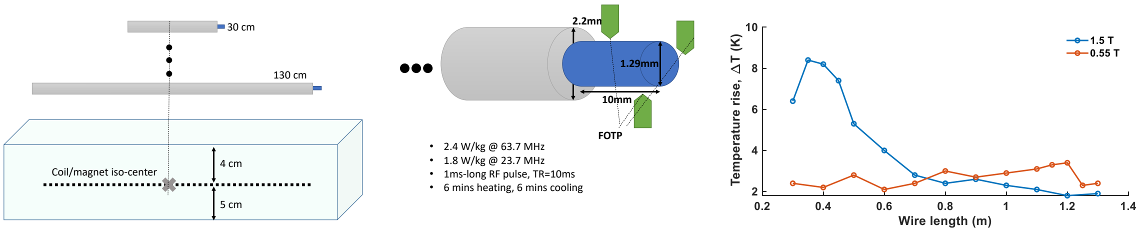

Supporting Information Figure S2: The resonance length at 23.7 MHz was measured to be 120 cm which is above typical device insertion lengths for all studied cases. For the measurements, an insulated wire with an exposed tip was used. At 1.5T, resonance length was measured as 35 cm.

Supporting Information Tables

Supporting Information Table S1: The results of the temperature measurements and TF-based temperature rise estimations.

Device	Position			ΔTmax (K)				Simulated ΔTmax (K)			
				Coil 1		Coil 2		Coil 1		Coil 2	
	z	x	y	157cm F	185cm M	157cm F	185cm M	157cm F	185cm M	157cm F	185cm M
GW	0	0	0	0.4	0.6	0.0	0.2	0.25	0.43	0.08	0.25
	0	5	0		0.7	0.1	0.2	0.15	0.34	0.15	0.22
	0	10	0	0.6	1.1	0.2	0.4	0.45	0.93	0.25	0.64
	0	-10	0	0.4	0.4	0.1	0.2	0.41	0.76	0.16	0.42
	-5	0	0	0.8	0.5	0.1	0.3	0.29	0.22	0.12	0.38
	-10	0	0	0.4	0.7	0.0	0.2	0.30	0.56	0.05	0.30
GC	0	0	0	0.2	0.3	0.0	0.0	0.05	0.07	0.09	0.12
	-10	0	0		0.2	0.0	0.0		0.08		0.18
GW	0	0	0	0.2	0.2	0.1	0.2	0.32	0.56	0.18	0.250
	0	10	0	0.4	0.4	0.1	0.1	0.30	0.40	0.17	0.132
	10	0	0	0.4	0.3	0.2	0.2	0.46	0.53	0.34	0.262
	-10	0	0	0.2	0.1	0.1	0.2	0.22	0.15	0.11	0.16
μC	0	0	0	0.5	0.6	0.2	0.4	0.78	1.43	0.21	0.78
	0	10	0	0.6	1.2	0.2	0.4	0.98	1.75	0.28	0.95
	10	0	0	0.6	0.6	0.2	0.4	0.81	0.96	0.24	0.55
	-10	0	0	0.4	0.2	0.1	0.2	0.40	0.80	0.12	0.62

Electromagnetic Simulation Settings

Simulation Settings	EM FDTD ASTM phantom
**Preparation**	
Type	*EM-FDTD Multi-port simulation*
**Setup**	
Simulation Time	40 Periods
Global Auto Termination	Medium
**Materials**	
Phantom/Gel	Dielectric
	er = 80
	s = 0.45 S/m
	c=1000kg/m3
Conductors	PEC
**Sources**	
Type	2xEdge port
Excitation signal	Gaussian
Frequency	23.7 MHz with 20 MHz span
Reference Load	Perfect matching assumed
**Lumped elements**	
**Element type**	**Inductor/ capacitor for the generic birdcage**
Calculated Self-Inductances	
Legs	267.70
Endrings	62.17
Calculated Effective Inductances	
Legs	153.87
Endrings	102.66
**Calculated Capacitances**	
Capacitance Value	30.11
Strength	Medium
**Grid**	306x306x120
	11.2 MCells
**Solver**	
Parallelization handling	Automatic
Kernel	CUDA
Solver mode	FDTD
**Analysis**	
Overall field	Simulation combiner
Tangential E field	Interpolation over line profiles extracted from the vascular models

3D models and capacitor values of a generic birdcage coil, phantom, vascular models and the Python scripts used in analysis of the simulation results can be downloaded from https://github.com/ozenEEE/UKF_SafeLowB0.
